# Proline-driven metabolic reprogramming promotes skeletal muscle hypertrophy and oxidative myofiber specification in porcine offspring: a stage-optimized maternal nutritional intervention

**DOI:** 10.1186/s40104-025-01232-7

**Published:** 2025-07-15

**Authors:** Jun Huang, Kaidi Ma, Junyi Wu, Shuangbo Huang, Zihao Huang, Yujiao Chen, Shijian Zhou, Hefeng Luo, Chengquan Tan

**Affiliations:** 1 State Key Laboratory of Swine and Poultry Breeding Industry, Guangdong Provincial Key Laboratory of Animal Nutrition Control, National Engineering Research Center for Breeding Swine Industry, College of Animal Science, South China Agricultural University, Guangzhou, 510642 China; 2 Dekon Food and Agriculture Group, Chengdu, 610225 China

**Keywords:** Mitochondrial function, Oxidative muscle fibers, Proline, Skeletal muscle development, STAT3 signaling pathway

## Abstract

**Background:**

While maternal proline (Pro) supplementation has demonstrated efficacy in enhancing placental angiogenesis and farrowing efficiency in swine, its regulatory role in fetal skeletal muscle ontogeny remains undefined. This study systematically evaluated the temporal-specific impacts of dietary Pro supplementation during critical phases of fetal myogenesis (encompassing primary myofiber formation and secondary myofiber hyperplasia) on offspring muscle development. A total of 120 sows with similar farrowing schedules were assigned to three groups: CON (basal diet), ST-Pro (0.5% Pro supplementation during secondary myofiber formation period, from d 60 gestation to farrowing), LT-Pro (0.5% Pro supplementation spanning primary and secondary myofiber formation period: from d 20 gestation to farrowing).

**Results:**

LT-Pro group significantly increased the longissimus dorsi (LD) muscle mass per unit body weight in newborn piglets compared to CON group (*P <* 0.05), while no such effect was observed in the ST-Pro group. Metabolomic profiling revealed elevated Pro, lysine, and tryptophan levels in the LD muscle of LT-Pro group piglets, accompanied by reduced branched-chain amino acids (BCAAs; leucine, isoleucine, and valine) in both serum and muscle (*P <* 0.05). Histological analysis demonstrated a 45.74% increase in myofiber cross-sectional area in the LT-Pro group (*P <* 0.05). At the molecular level, LT-Pro group piglets exhibited upregulated mRNA expression levels of myogenic regulatory genes (*MYOD1*, *MYF6*) and the cell cycle accelerator *CCND1* (*P <* 0.05), coupled with activation of the STAT3 signaling pathway (phosphorylated STAT3 protein increased by 2.53-fold, *P <* 0.01). Furthermore, Pro supplementation enhanced oxidative metabolism, evidenced by elevated mitochondrial biogenesis markers (the mRNA expression levels of *PPARGC1A*, *OPA1*, and *SQSTM1*) and a 61.58% increase in succinate dehydrogenase activity (*P <* 0.05). Notably, LT-Pro group piglets showed a selective shift toward slow-twitch oxidative fibers, with both MyHC1 mRNA and protein expression levels significantly upregulated (*P* < 0.05), while the mRNA expression levels of *MyHCIIb* showed no significant change.

**Conclusions:**

This study identified the primary fiber formation period as a critical window. Supplementation with Pro during G20–114 reprogrammed offspring skeletal muscle development through STAT3-CCND1-mediated myoblast proliferation, enhanced mitochondrial bioenergetics, and oxidative fiber specification. However, no such effects were observed during G60–114. These findings propose maternal Pro intervention as a novel strategy to enhance muscle yield and metabolic efficiency in swine production, with potential applications for improving meat quality traits linked to oxidative muscle phenotypes.

**Graphical Abstract:**

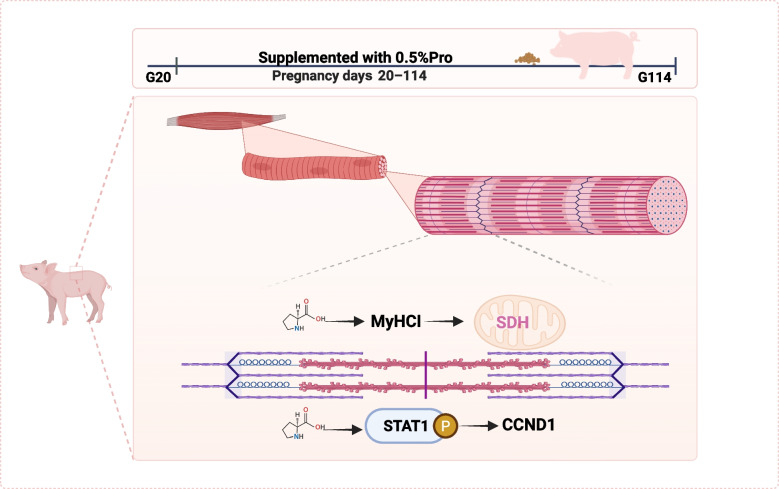

**Supplementary Information:**

The online version contains supplementary material available at 10.1186/s40104-025-01232-7.

## Introduction

Skeletal muscle development in mammals is a highly regulated biological process, governed by both genetic programming and maternal nutritional status during critical periods of fetal growth [1, 2]. In livestock production, optimizing maternal nutrition to enhance offspring muscle growth is essential for improving both meat yield and quality [3]. Among various nutrients, amino acids play a key role in fetal programming, with proline (Pro) being a particularly multifunctional amino acid involved in collagen synthesis, redox homeostasis, and cell signaling [4–6]. Previous studies conducted by our research group have demonstrated that maternal Pro supplementation improves placental angiogenesis and offspring survival rates in pigs [7]; however, its specific impact on muscle development and muscle fiber formation in offspring remains largely unexplored. Given the economic significance of skeletal muscle mass in pork production, understanding how Pro influences muscle growth is crucial.

Fetal muscle development in pigs follows two distinct stages: primary fiber formation (gestational d 20–60) and secondary fiber proliferation (gestational d 60–90) [8, 9]. These developmental windows represent sensitive periods during which nutritional interventions can have lasting epigenetic and metabolic effects on muscle phenotype. While the role of branched-chain amino acids (BCAAs) in promoting muscle growth and protein synthesis has been widely studied, the effects of Pro on the regulation of myogenic transcription factors and muscle fiber type differentiation have not been well clarified [10–13]. This gap in knowledge is particularly intriguing, as Pro acts not only as a metabolic substrate but also as a signaling molecule capable of activating nutrient-sensitive pathways involved in muscle growth and fiber type.

In this study, we hypothesize that maternal Pro supplementation during the primary fiber development period (G20–114) will differentially regulate offspring skeletal muscle development compared to supplementation during the secondary fiber development period (G60–114). Specifically, we aim to investigate whether Pro supplementation during the critical G20–114 period influences myogenic gene expression, muscle fiber composition, and mitochondrial bioenergetics, which are essential for muscle growth and functional development. By addressing this gap, our findings could provide new insights into optimizing maternal nutrition strategies for improving muscle growth and meat quality in swine, thereby contributing to more efficient pork production.

## Materials and methods

### Animal ethics statement

This study was conducted in a commercial research facility at Zhaoqing Baoyuan Agriculture Co., Ltd. The gilts used in the experiments were humanely treated following the practices outlined in the Guide for the Animal Ethics Committee of South China Agricultural University (2023G017).

### Experimental design, diets, and data collection

On gestational d 20, a total of 120 primiparous sows (Landrace × Yorkshire) with similar backfat thickness were randomly assigned to three treatment groups, with 40 sows per group and each sow serving as an individual replicate. Three sows were removed from the trial prior to farrowing due to severe lameness or mortality, resulting in 117 sows completing the experiment and being included in the final data analysis. The effective replicates for the control group (CON), ST-Pro group (Short-term addition), and LT-Pro group (Long-term addition) were 38, 40, and 39, respectively. The trial spanned from gestational d 20 to 114. Two experimental diets were formulated for this study: a basal diet or a basal diet supplemented with 0.5% Pro. The Pro levels were selected based on previous studies showing that dietary supplemented with 0.5% Pro optimizes placental function without exceeding the safe upper limit for sow metabolism [7]. Notably, both diets were iso-nitrogenous. Sows in the CON group were fed the basal diet. Sows in the ST-Pro group received the basal diet from gestational d 20 to 59, and were switched to the test diet from d 60 to 114. Sows in the LT-Pro group were fed the test diet throughout the entire experimental period (d 20 to 114). The ingredient composition and nutritional levels of the basal and test diets are detailed in Table S1. Sows were housed individually and provided a constant daily feed allowance of 2.5 kg during gestation. Postpartum data, including the number of live-born piglets, normal-weight piglets, low-birth-weight piglets, stillbirths, malformed fetuses, mummified fetuses, and non-viable fetuses, as well as their body weights, were recorded [7].

All experimental diets were formulated to meet the nutrient requirements for sows [14]. Chemical concentrations were calculated using the values for feed ingredients from the National Research Council [14]. The contents of crude protein, crude fiber and neutral detergent fiber in the experimental diet were analyzed according to the National Standards of the People’s Republic of China of GB/T 6432-2018 [15], GB/T 6434-2022 [16] and GB/T 20806-2022 [17], respectively.

### Sample collection

On parturition day, 8 newborn piglets were randomly selected from each group (each piglet corresponded to a different sow), and blood samples were collected from the anterior vena cava of newborn piglets using 10-mL centrifuge tubes, and then centrifuged at 3,000 × *g* and 4 °C for 15 min to recover serum. After euthanasia by intravenous injection of sodium pentobarbital, two samples of each of the longissimus dorsi (LD), soleus, and psoas major (PM) were collected from the left side of the piglets, one of which was snap-frozen in liquid nitrogen and the other was fixed in 4% paraformaldehyde. The ileal mucosa of the piglets was collected and stored at −80 °C for further analysis.

### Targeted metabolomics assays

Amino acids from piglet serum, and LD muscle were measured by using high-performance liquid chromatography-tandem mass spectrometry (HPLC-MS/MS). Metabolites were extracted according to standard methods [18]. Briefly, 200 μL of piglet serum and LD muscle homogenate were mixed with 800 μL of acetonitrile, respectively. The samples were then homogenized, sonicated, centrifuged (12,500 × *g*, 4 °C, 15 min), dried, and re-dissolved, and 200 μL of the supernatant was used for HPLC-MS/MS analysis. Data analysis was performed using Xcalibur 4.7 (Thermo, USA).

### Muscle morphology observation

LD muscle fixed in 4% paraformaldehyde was paraffin-embedded and sectioned at 5 μm thickness, followed by staining with hematoxylin-eosin (HE). Four fascicles were randomly selected from each section and used for image analysis using a light microscope (Olympus CX41, Tokyo, Japan). ImageJ software was used to count the total muscle fiber cross-sectional area and number in the field of view, and the cross-sectional area of each muscle fiber was obtained by dividing the total area by the number.

### Measurement of mitochondrial DNA (mtDNA)

Total DNA was isolated from 200 mg of LD muscle using the Qiagen DNA Mini Kit (51306, Qiagen, Germany). Then, mtDNA was amplified using primers specific for the mitochondrial cytochrome b (forward: 5′-ATGAAACATTGGAGTAGTCCTACTATTTACC-3′; reverse: 5′-CTACGAGGTCTGTTCCGATATAAGG-3′), and normalized to genomic DNA by amplification of the 18S ribosomal RNA (forward: 5′-GGTAGTGACGAAAAATAACAATACAGGAC-3′; reverse: 5′-ATACGCTATTGGAGCTGGAATTACC-3′).

### Biochemical analysis

Approximately 200 mg of LD muscle samples were completely homogenized in saline and centrifuged at 12,000 × *g* for 5 min at 4 °C. The supernatant was then isolated for subsequent biochemical analysis. The levels of adenosine triphosphate (ATP, S0026, Beyotime, China), and succinate dehydrogenase (SDH, BC0950, Solarbio, China) were measured using commercial kits. Results were normalized to total protein (P0009, Beyotime, China) or weight.

### RT-qPCR analysis of gene expression

According to the manufacturer’s instructions, the total muscle and ileal mucosa RNA was extracted using the RNA extraction kit (EZBioscience, Guangzhou, China). The A_260_/A_280_ ratio of the RNA used for the experiment should be between 1.8 and 2.0. After reverse transcription using PrimerScript RT reagent Kit (EZBioscience, Guangzhou, China), RT-qPCR was performed to analyze the expression levels of related genes on a QuantStudio 6 RealTime PCR System (Thermo Fisher, Waltham, USA). The relative expression was calculated using the comparative method (2^-ΔΔCt^), with β-actin as the internal control. The primers used in the experiments are listed in Table S2.

### Western blotting

Total proteins were extracted from 200 mg of LD muscle using a protein extraction kit (NCM, China). Then, protein concentration was detected using bicinchoninic acid protein assay kits (P0009, Beyotime, China). After being separated by sodium dodecyl sulfate-polyacrylamide gel electrophoresis, proteins were transferred onto a polyvinylidene difluoride membrane (Merck Millipore) and blocked with bovine serum albumin Tris-Tween-buffered saline buffer. The membranes were then incubated with primary antibodies [signal transducer and activator of transcription 3 (STAT3, ab76315, Abcam, USA, 1:1,500), p-STAT3 (ab76315, Abcam, USA, 1:1,500), Myosin Heavy Chain I (MyHCI, DSHB, USA, 1:1,000), and β-Actin (4970, CST, USA, 1:1,000)] and secondary antibodies (HPR Goat Anti-Rabbit, AS028, ABclonal, China; Goat Anti-Mouse, ab205719, USA) successively. Finally, images were captured using the ChemiDoc MP system (Bio-Rad, Hercules, CA, USA), and band densities were quantified using Image Lab software (Bio-Rad, Hercules, CA, USA) and then normalized to β-actin content.

### Statistical analysis

All data are expressed as mean ± standard error of the mean (SEM). Statistical analyses were conducted using one-way analysis of variance (ANOVA) in GraphPad Prism (version 10.1.2). Tukey’s multiple comparisons test was applied as a post hoc analysis to identify specific group differences. Differences between groups were considered statistically significant at *P* < 0.05.

## Results

### Sow reproductive performance and muscle mass of newborn piglets

No significant differences were observed in birth weight among the three treatment groups (*P* = 0.70) (Table 1). Similarly, the weights of the psoas major (*P* = 0.50) and soleus muscles (*P* = 0.31) did not differ significantly across groups.

**Table 1:** Newborn piglets' muscle mass^1^

Item	CON	ST-Pro	LT-Pro	SEM	*P*-value
Birth weight of piglet, g	1,469.13	1,436.88	1,451.00	15.11	0.70
PM muscle, g	2.77	3.13	3.15	0.14	0.50
Soleus, g	0.87	0.88	0.97	0.03	0.31
LD muscle, g	11.07^b^	13.68^ab^	15.42^a^	0.65	< 0.05
Back muscle weight per unit body weight, g/kg	7.50^b^	9.53^ab^	10.59^a^	0.42	< 0.05

^1^*Pro *Proline, *PM muscle *Psoas major muscle, *LD muscle * Longissimus dorsi muscle, *CON *The group fed a basal diet, *ST-Pro *Short-term addition, basal diet supplemented with 0.5% Pro from G60 until farrowing, *LT-Pro *Long-term addition, basal diet supplemented with 0.5% Pro from G20 until farrowing

^a,b^Different lowercase letters in each row represent significant difference at *P* < 0.05; *SEM * Standard error of the mean, *n* = 8

However, significant effects were observed on the LD muscle weight and LD muscle weight per unit body weight. The LD muscle weight in the LT-Pro group (15.42 g) was significantly higher than that in the CON group (11.07 g; *P* < 0.05), while the ST-Pro group (13.68 g) showed no statistically significant differences compared to the other two groups. Similarly, the LD muscle weight per unit body weight in the LT-Pro group (10.59 g/kg) was significantly greater than that in the CON group (7.50 g/kg; *P* < 0.05), with the ST-Pro group (9.53 g/kg) exhibiting no significant differences compared to the other two groups.

### Amino acid metabolic concentrations in the serum and LD muscle of newborn piglets

In serum (Table 2), significant differences were observed for asparagine (Asn), aspartic acid (Asp), glutamic acid (Glu), valine (Val), isoleucine (Ile), and methionine (Met) (*P <* 0.05). The LT-Pro group exhibited the highest concentrations of Asn (3.45 µg/mL), Asp (0.55 µg/mL), and Glu (4.69 µg/mL), which were significantly higher than those in the CON group (Asn: 1.98 µg/mL; Asp: 0.42 µg/mL; Glu: 3.59 µg/mL) (*P <* 0.05). In contrast, the concentrations of Val and Ile were significantly lower in the LT-Pro group (Val: 30.31 µg/mL; Ile: 13.59 µg/mL) compared to the CON group (Val: 43.75 µg/mL; Ile: 23.19 µg/mL) (*P <* 0.05). The Met concentrations in the ST-Pro and LT-Pro groups (1.39 µg/mL and 1.42 µg/mL, respectively) tended to be lower than that in the CON group (2.32 µg/mL) (*P* = 0.05). Additionally, the histidine (His) concentration in the LT-Pro group (0.41 µg/mL) tended to be higher than that in the CON and ST-Pro groups (0.32 µg/mL) (*P* = 0.06). Similarly, the Pro concentration in the LT-Pro group (12.13 µg/mL) tended to be higher than that in the CON group (7.41 µg/mL) and the ST-Pro group (8.69 µg/mL) (*P* = 0.06). No significant differences were observed for other amino acids, including arginine (Arg), lysine (Lys), glycine (Gly), serine (Ser), alanine (Ala), glutamine (Gln), threonine (Thr), tyrosine (Tyr), leucine (Leu), phenylalanine (Phe), and tryptophan (Trp) (*P* > 0.10).

**Table 2:** Amino acid concentrations in serum of newborn piglets^1^

Item	CON	ST-Pro	LT-Pro	SEM	*P*-value
Number of repeats	8	8	8		
Serum, µg/mL					
Ala	4.34	3.81	5.03	0.25	0.12
Arg	0.65	0.72	0.54	0.06	0.44
Asn	1.98^b^	2.14^b^	3.45^a^	0.24	<0.05
Asp	0.42^b^	0.42^b^	0.55^a^	0.02	<0.01
Gln	1.07	0.85	1.03	0.06	0.29
Glu	3.59^b^	3.67^b^	4.69^a^	0.18	<0.05
Gly	1.55	1.38	1.58	0.05	0.16
His	0.32	0.32	0.41	0.02	0.06
Ile	23.19^b^	20.46^ab^	13.59^b^	1.63	<0.05
Leu	16.12	14.22	10.69	1.09	0.12
Lys	0.76	0.84	0.70	0.10	0.85
Met	2.32	1.39	1.42	0.18	0.05
Phe	10.42	8.74	9.68	0.52	0.44
Pro	7.41	8.69	12.13	0.87	0.06
Ser	1.20	1.34	1.51	0.07	0.24
Thr	1.20	1.17	1.13	0.14	0.98
Trp	5.38	6.16	6.60	0.36	0.40
Tyr	8.48	8.62	9.24	0.85	0.93
Val	43.75^a^	31.47^b^	30.31^b^	1.91	<0.01

^1^*CON* The group fed a basal diet, *ST-Pro *Short-term addition, basal diet supplemented with 0.5% Pro from G60 until farrowing, *LT-Pro *Long-term addition, basal diet supplemented with 0.5% Pro from G20 until farrowing

^a,b^Different lowercase letters in each row represent significant difference at *P* < 0.05; *SEM * Standard error of the mean

In the LD muscle (Table 3), the concentrations of Lys, Pro, Ile, Leu, and Trp were significantly influenced by maternal Pro supplementation (*P <* 0.05). Specifically, the LT-Pro group showed the highest concentrations of Lys (1.15 µg/mL) and Pro (11.00 µg/mL), which were significantly higher than those in the CON group (Lys: 0.83 µg/mL; Pro: 5.80 µg/mL) (*P <* 0.05). Conversely, the concentrations of Ile and Leu were significantly lower in the LT-Pro group (Ile: 3.53 µg/mL; Leu: 2.45 µg/mL) compared to the CON group (Ile: 5.13 µg/mL; Leu: 3.57 µg/mL) (*P <* 0.01). The Trp concentration was also significantly higher in the LT-Pro group (0.88 µg/mL) compared to the CON and ST-Pro groups (0.70 µg/mL and 0.67 µg/mL, respectively) (*P <* 0.01). The Arg concentrations in the CON and LT-Pro groups (0.30 µg/mL and 0.32 µg/mL, respectively) tended to be lower than that in the ST-Pro group (0.38 µg/mL) (*P* = 0.06). Additionally, the Asn concentration in the LT-Pro group (3.14 µg/mL) tended to be higher than that in the CON and ST-Pro groups (2.53 µg/mL and 2.70 µg/mL, respectively) (*P* = 0.06). Similarly, the Tyr concentration in the LT-Pro group (1.44 µg/mL) tended to be higher than that in the CON group (1.17 µg/mL) and the ST-Pro group (1.02 µg/mL) (*P* = 0.08). No significant differences were observed for other amino acids, including His, Gly, Ser, Asp, Ala, Gln, Thr, Glu, Val, Met, and Phe (*P* > 0.10).

**Table 3:** Amino acid concentrations in the LD muscle of newborn piglets^1^

Item	CON	ST-Pro	LT-Pro	SEM	*P*-value
Number of repeats	8	8	8		
LD muscle, µg/mL					
Ala	21.21	23.48	21.06	0.53	0.11
Arg	0.30	0.38	0.32	0.01	0.06
Asn	2.53	2.70	3.14	0.11	0.06
Asp	3.33	2.62	2.76	0.17	0.19
Gln	5.77	7.10	6.99	0.36	0.25
Glu	39.84	44.50	46.92	2.07	0.38
Gly	3.02	2.75	2.60	0.17	0.63
His	1.05	1.01	0.92	0.05	0.56
Ile	5.13^a^	4.21^ab^	3.53^b^	0.22	<0.01
Leu	3.57^a^	2.93^ab^	2.45^b^	0.15	<0.01
Lys	0.83^b^	1.00^ab^	1.15^a^	0.05	<0.05
Met	0.44	0.54	0.39	0.03	0.14
Phe	1.48	1.31	1.40	0.05	0.43
Pro	5.80^b^	7.04^ab^	11.00^a^	0.85	<0.05
Ser	3.93	4.76	4.78	0.23	0.23
Thr	1.80	1.66	2.12	0.13	0.36
Trp	0.70^b^	0.67^b^	0.88^a^	0.03	<0.01
Tyr	1.17	1.02	1.44	0.08	0.08
Val	21.70	19.63	16.82	1.18	0.24

^1^*CON * The group fed a basal diet, *ST-Pro *Short-term addition, basal diet supplemented with 0.5% Pro from G60 until farrowing, *LT-Pro* Long-term addition, basal diet supplemented with 0.5% Pro from G20 until farrowing

^a,b^Different lowercase letters in each row represent significant difference at *P* < 0.05; *SEM *Standard error of the mean

### LD muscle fiber characteristics of newborn piglets

**Fig. 1:**
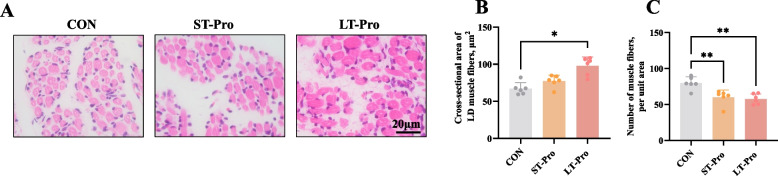
LD muscle fiber characteristics of newborn piglets. **A** Hematoxylin-eosin (HE) staining to show the muscle fiber. Bar = 20 μm. **B** The cross-sectional area of each muscle fiber of the longissimus dorsi. **C** The number of muscle fibers per unit area of the longissimus dorsi. Data were expressed as means ± SEM, *n* = 6 (four fields of view were randomly captured for each sample). ** and * represent *P* < 0.01 and *P* < 0.05 respectively. CON: The group fed a basal diet; ST-Pro: Short-term addition, basal diet supplemented with 0.5% Pro from G60 until farrowing; LT-Pro: Long-term addition, basal diet supplemented with 0.5% Pro from G20 until farrowing

To determine whether dietary treatment was mediated by changes in muscle fiber morphology, we evaluated the cross-sectional area (CSA) of LD muscle fibers using HE staining. The results (Fig. 1A–C) indicated that LT-Pro group significantly increased the CSA of LD muscle fibers in newborn piglets by 45.74% compared to the CON group (*P <* 0.05). However, ST-Pro group did not significantly affect the CSA of LD muscle fibers. Correspondingly, LT-Pro group significantly reduced the number of muscle fibers per unit area (*P <* 0.05), consistent with the observed increase in fiber CSA.

### Expression of amino acid transporter genes in the LD muscle and intestinal mucosa of newborn piglets

To elucidate the mechanisms underlying Pro-induced muscle development, we analyzed the expression of amino acid transporter genes in the LD muscle and ileal mucosa of newborn piglets. These transporters mediate nutrient uptake and distribution, potentially serving as key regulators of maternal nutrient programming on offspring muscle biology.

**Fig. 2:**
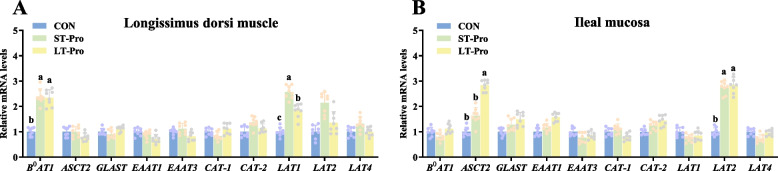
Expression of amino acid transporter genes in the intestinal mucosa and LD muscle of newborn piglets. **A** Expression of amino acid transporter mRNA in longissimus dorsi muscle of newborn piglets. **B** Expression of amino acid transporter mRNA in the ileal mucosa of newborn piglets. Data were presented as means ± SEM, *n* = 8. ^a,b,c^Different lowercase letters represent significant difference at *P* < 0.05. CON: The group fed a basal diet; ST-Pro: Short-term addition, basal diet supplemented with 0.5% Pro from G60 until farrowing; LT-Pro: Long-term addition, basal diet supplemented with 0.5% Pro from G20 until farrowing

In the LD muscle of newborn piglets (Fig. 2A), LT-Pro group significantly upregulated the mRNA expression of the neutral amino acid transporter *B*^*0*^*AT1* and the branched-chain amino acid transporter *LAT1* compared to the control group (*P <* 0.05). Similarly, ST-Pro group increased the mRNA expression of both *B*^*0*^*AT1* and *LAT1* (*P <* 0.05). However, Pro supplementation at either stage showed no significant effects on the mRNA expression of other transporters, including neutral (*ASCT2*), acidic (*GLAST, EAAT1, EAAT3*), basic (*CAT-1, CAT-2*), or additional BCAA transporters (*LAT2, LAT4*) (*P* > 0.05).

In the ileal mucosa (Fig. 2B), LT-Pro group significantly enhanced the mRNA expression of the neutral transporter *ASCT2* and BCAA transporter *LAT2* (*P <* 0.05). ST-Pro group significantly upregulated the mRNA expression of *LAT2* (*P <* 0.05). No significant changes were observed in the mRNA expression of neutral (*B*^*0*^*AT1*), acidic (*GLAST*, *EAAT1*, *EAAT3*), basic (*CAT-1*, *CAT-2*), or other BCAA transporters (*LAT1*, *LAT4*) across treatment groups (*P* > 0.05).

### Expression of muscle development-related genes in the LD muscle of newborn piglets

To investigate the molecular mechanisms underlying Pro-mediated muscle development, we evaluated the expression of key genes regulating myogenesis, satellite cell activation, and cell cycle progression in the LD muscle. These genes were selected based on their established roles in muscle biology: *MYOD1* (myoblast differentiation), *MYF6* (myofiber maturation), *CCND1* (cell cycle regulation), *MYOG* (terminal differentiation), *IGF1* (growth factor signaling), *PAX7* (satellite cell maintenance), *MYF5* (myogenic commitment), *MEF2C/MEF2D* (fiber type specification), *FGF2* (proliferation), *TCF4*/*SIX1* (embryonic myogenesis), *CCND2*/*CCNB1* (cell cycle phases), and *MSTN* (muscle growth inhibition) [19–23].

**Fig. 3:**
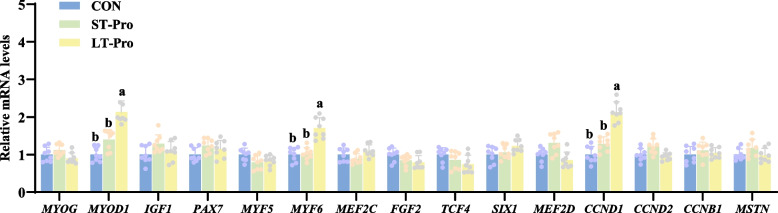
Expression of muscle development-related genes in the LD muscle of newborn piglets. Data were presented as means ± SEM, *n* = 8. ^a,b^Different lowercase letters represent significant difference at *P* < 0.05. CON: The group fed a basal diet; ST-Pro: Short-term addition, basal diet supplemented with 0.5% Pro from G60 until farrowing; LT-Pro: Long-term addition, basal diet supplemented with 0.5% Pro from G20 until farrowing

As shown in Fig. 3, LT-Pro group significantly upregulated the mRNA expression of critical myogenic regulators in the LD muscle of newborn piglets compared to the CON group (*P <* 0.05). Specifically: *MYOD1*, a master transcription factor governing myoblast differentiation, increased by 2.13-fold. *MYF6* (also known as *MRF4*), essential for myofiber maturation, exhibited a 1.71-fold elevation. *CCND1*, a cell cycle accelerator driving myoblast proliferation, rose by 2.14-fold. In contrast, no significant changes were observed in the mRNA expression of other genes involved in muscle development, including *MYOG*, *IGF1*, *PAX7*, *MYF5*, *MEF2C*, *FGF2*, *TCF4*, *SIX1*, *MEF2D*, *CCND2*, *CCNB1*, or *MSTN* (*P* > 0.05). Notably, ST-Pro group had no significant effects on the expression of any tested genes (*P* > 0.05).

### Muscle fiber types in the LD muscle of newborn piglets

To assess Pro’s impact on meat quality-related fiber typing, we analyzed myosin heavy chain (MyHC) isoforms in the LD muscle. MyHCI (slow-twitch oxidative fiber marker) and MyHCII isoforms (IIa, IIx, IIb; fast-twitch glycolytic fiber markers) were analyzed at both transcriptional (qPCR) and translational (Western blot) levels to ensure comprehensive assessment.

**Fig. 4:**
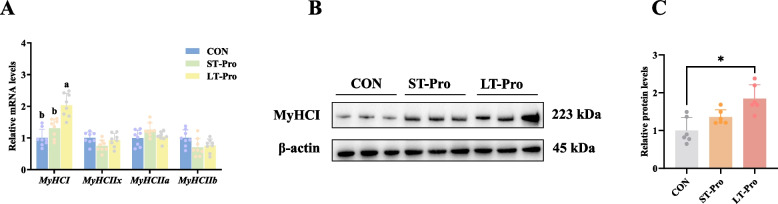
Muscle fiber types in the LD muscle of newborn piglets. **A** mRNA expression of genes related to muscle fiber type in longissimus dorsi muscle of newborn piglets (*n* = 8). **B** Immunoblotting of MyHCI in the longissimus dorsi muscle of newborn piglets (*n* = 6). **C** Quantification of MyHCI. Data were presented as means ± SEM. ^a,b^Different lowercase letters represent significant difference at *P* < 0.05 and * represents *P* < 0.05. CON: The group fed a basal diet; ST-Pro: Short-term addition, basal diet supplemented with 0.5% Pro from G60 until farrowing; LT-Pro: Long-term addition, basal diet supplemented with 0.5% Pro from G20 until farrowing

As shown in Fig. 4A, qPCR analysis revealed that LT-Pro group significantly upregulated *MyHCI* gene expression in the LD muscle of newborn piglets compared to the CON group (*P <* 0.05). In contrast, no significant differences were observed in the mRNA expression of *MyHCIIx*, *MyHCIIa*, or *MyHCIIb* (*P* > 0.05). ST-Pro group had no significant effect on the expression of any *MyHC* genes (*P* > 0.05).

Western blot results (Fig. 4B and C) corroborated the transcriptional findings, demonstrating a significant increase in MyHCI protein abundance in the LT-Pro group compared to the CON group (*P <* 0.05). Consistent with the qPCR data, ST-Pro group did not alter MyHCI protein levels (*P* > 0.05).

### Mitochondrial function in the LD muscle of newborn piglets

**Fig. 5:**
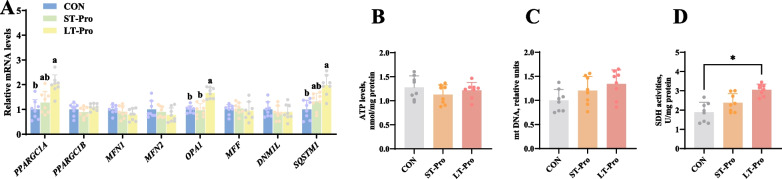
Mitochondrial function in the LD muscle of newborn piglets. **A** Expression results of genes related to mitochondrial dynamics in the longissimus dorsi muscle. **B** SDH activity of the longissimus dorsi muscle. **C** mtDNA content in the longissimus dorsi muscle. **D** ATP levels in the longissimus dorsi muscle. Data were presented as means ± SEM, *n* = 8. ^a,b^Different lowercase letters represent significant difference at *P* < 0.05 and * represents *P* < 0.05. CON: The group fed a basal diet; ST-Pro: Short-term addition, basal diet supplemented with 0.5% Pro from G60 until farrowing; LT-Pro: Long-term addition, basal diet supplemented with 0.5% Pro from G20 until farrowing

To evaluate the impact of maternal Pro supplementation on offspring muscle mitochondrial function, we analyzed both molecular markers of mitochondrial dynamics and key biochemical indicators of energy metabolism. Figure 5A shows the gene expression results related to mitochondrial dynamics. qPCR analysis revealed that LT-Pro group significantly upregulated the mRNA expression of mitochondrial regulators *PPARGC1A* (2.01-fold), *OPA1* (1.67-fold), and *SQSTM1* (1.98-fold) in the LD muscle of newborn piglets compared to the CON group (*P <* 0.05). In contrast, no significant changes were observed in the mRNA expression of *PPARGC1B*, *MFN1*, *MFN2*, *MFF*, or *DNM1L* (*P* > 0.05). ST-Pro group had no significant effect on the expression of any tested mitochondrial genes (*P* > 0.05).

Figure 5B–D shows mitochondrial function indicators. Biochemical assays demonstrated that LT-Pro group significantly enhanced SDH activity in the LD muscle by 61.58% compared to the CON group (*P <* 0.05). However, no significant differences were observed in mtDNA content or ATP levels across all groups (*P* > 0.05). Consistent with the gene expression findings, ST-Pro group showed no significant effects on mtDNA content, ATP levels, or SDH activity (*P* > 0.05).

These results collectively suggest that the LT-Pro group may enhance mitochondrial function in offspring skeletal muscle, potentially through selective upregulation of *PPARGC1A*-mediated mitochondrial biogenesis and *SQSTM1*-dependent mitophagy, accompanied by improved SDH-driven oxidative capacity.

### STAT3 protein expression in the LD muscle of newborn piglets

**Fig. 6:**
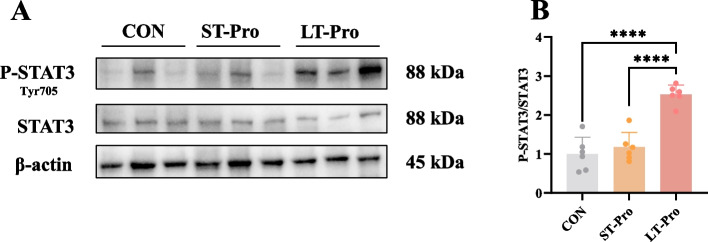
STAT3 protein expression in the LD muscle of newborn piglets. **A** Immunoblotting of p-STAT3 and STAT3 in the longissimus dorsi muscle of newborn piglets. **B** Quantification of p-STAT3/STAT3. Data were presented as means ± SEM, *n* = 6. **** represents *P* < 0.0001. CON: The group fed a basal diet; ST-Pro: Short-term addition, basal diet supplemented with 0.5% Pro from G60 until farrowing; LT-Pro: Long-term addition, basal diet supplemented with 0.5% Pro from G20 until farrowing

To determine whether Pro supplementation at different stages of pregnancy would affect the muscle development of offspring through STAT3, we detected the expression of STAT3 protein. Western blot analysis (Fig. 6A and B) demonstrated that LT-Pro group significantly increased the abundance of P-STAT3 protein in the LD muscle of newborn piglets compared to the CON group (*P <* 0.05), indicating activation of the STAT3 signaling pathway. In contrast, ST-Pro group had no significant effect on P-STAT3 levels (*P* > 0.05).

Integration of these findings with the qPCR data (Fig. 3)—which showed upregulated *CCND1* expression in the LT-Pro group—suggests that maternal Pro supplementation promotes LD muscle proliferation through the STAT3/CCND1 pathway. This mechanistic link likely contributes to the observed increase in LD muscle mass in G20-114 offspring.

## Discussion

This study establishes maternal Pro supplementation from the primary myogenesis window (G20–114) as a novel nutritional strategy to enhance offspring skeletal muscle development in swine. By integrating multi-level analyses—from amino acid metabolism and mitochondrial bioenergetics to myogenic programming—we identify three interlinked mechanisms underpinning Pro’s developmental reprogramming effects. These findings offer transformative insights into prenatal nutrient timing and muscle biology.

### Temporal-specific nutritional programming: a window of developmental sensitivity

The selective enhancement of LD muscle mass and myofiber hypertrophy in the LT-Pro group (Table 1 and Fig. 1) underscores the unique sensitivity of primary myogenesis to maternal Pro availability. The G20–114 window coincides with the peak of primary myofiber formation in pigs. During this period, muscle progenitor cells demonstrate heightened sensitivity to nutrient availability, undergoing fate determination and rapid proliferation [24]. This period precedes satellite cell differentiation into secondary fibers, explaining the lack of ST-Pro group effects despite identical Pro dosage. The absence of similar effects in the ST-Pro group—despite comparable Pro intake—highlights the irreversibility of primary myofiber patterning after this critical window.

The differential regulation of intestinal amino acid transporters further supports this temporal specificity (Fig. 2). ST-Pro group upregulated the mRNA expression of *LAT2* (a BCAA-preferring transporter [10]), while LT-Pro group enhanced the mRNA expression of *ASCT2*, promoting the uptake of glutamine and neutral amino acids, a process critical for mTOR activation [25–27]. This divergence reflects stage-specific nutrient priorities: early gestation prioritizes substrates for hyperplastic growth (e.g., glutamine for nucleotide synthesis), whereas late gestation favors hypertrophic expansion via BCAA-driven mTOR signaling [28]. Our findings align with developmental biology models showing that nutrient availability during lineage commitment phases exerts lifelong phenotypic effects [29–32], but extend this paradigm to amino acid-specific programming.

### Metabolic reprogramming: proline as a nexus of anabolism and oxidative metabolism

The Pro-induced metabolic shift (Table 2 and Fig. 4) reveals a previously unrecognized competition between Pro and BCAAs in prenatal muscle development. Elevated muscle Pro, Lys, and Trp (Table 2) likely synergize to promote collagen crosslinking and serotonin synthesis—both essential for extracellular matrix remodeling during myotube formation [33–35]. Concurrently, the depletion of BCAAs (leucine, isoleucine, valine) suggests their accelerated catabolism to fuel tricarboxylic acid (TCA) cycle intermediates, as evidenced by increased SDH activity (Fig. 4). This creates a “pro-anabolic” milieu where Pro-derived glutamate (serum elevation in Table 2) enters the purine nucleotide cycle to support myoblast proliferation [36–39]. The upregulation of *ASCT2* in the ileal mucosa (Fig. 2) likely enhances glutamine uptake, which serves as a precursor for glutamate synthesis. This may synergize with Pro-derived glutamate (Table 2) to activate STAT3 signaling, thereby linking intestinal nutrient transport to myogenic programming.

The coordinated upregulation of *B*^*0*^*AT1* (Fig. 2)—a sodium-coupled neutral amino acid transporter—ensures efficient Pro uptake into myocytes, while *PPARGC1A* and *OPA1* induction (Fig. 4) enhances mitochondrial fusion and oxidative capacity. Notably, the selective increase in MyHCI expression (Fig. 5) aligns with this metabolic rewiring, as oxidative fibers rely heavily on mitochondrial ATP production [40, 41].

### STAT3-CCND1 axis: bridging nutrient signaling and myogenic transcription

The activation of STAT3 phosphorylation (Fig. 6) provides a direct mechanistic link between maternal Pro supplementation and enhanced myogenesis. STAT3 is a nutrient-sensitive transcription factor that binds the *CCND1* promoter to drive myoblast proliferation [42–46], creating a feed-forward loop amplified by Pro’s effects during primary myogenesis (Fig. 3). Our data suggest that Pro not only activates STAT3 but also sustains *CCND1* expression through transcriptional regulation—a mechanism consistent with the observed upregulation of myogenic regulators (*MYOD1*, *MYF6*) and oxidative fiber markers (*PPARGC1A*, *MyHCI*).

The STAT3-*PPARGC1A* cross-talk (Figs. 4 and 5) further explains the oxidative fiber specification. STAT3 physically interacts with *PPARGC1A* to co-activate slow-twitch fiber genes [47, 48], a mechanism likely reinforced by Pro’s mitochondrial enhancements (e.g., SDH activity). This dual role of STAT3—orchestrating both proliferation (via *CCND1*) and oxidative metabolism (via * PPARGC1A*)—positions it as a central hub for Pro-mediated muscle programming.

Our results suggest that Pro could be established as a new prenatal programming agent. This paradigm shift is supported by three interrelated observations. First, maternal Pro supplementation induced an inverse relationship between Pro and BCAA levels in offspring muscle and serum (Table 2), suggesting Pro redirects metabolic flux away from BCAA-driven anabolism toward oxidative pathways. Second, Pro selectively enhanced oxidative fiber development (MyHCI upregulation, Fig. 5) without affecting glycolytic isoforms (MyHCIIb), contrasting sharply with BCAA’s preferential promotion of fast-twitch hypertrophy in postnatal models [49]. Third, the mitochondrial remodeling observed here—marked by *PPARGC1A* induction and SDH activation (Fig. 4)—diverges fundamentally from the mTOR-centric mechanisms typically associated with BCAA supplementation. These distinctions underscore the temporal specificity of nutrient actions: while BCAAs dominate postnatal growth via mTOR [50–52], Pro primes prenatal muscle for oxidative efficiency through mitochondrial and epigenetic adaptations.

The G20–114 window identified here challenges the industry’s focus on late-gestation “flushing” strategies. Previous studies from our research group have shown that adding sufficient Pro (1.39% to 1.89%) to the diet of sows can increase the average birth weight of piglets, litter weight, and the number of normal birth weight piglets, while reducing the number of mummified fetuses, the rate of low birth weight, and the rate of non-viable piglets [7]. Therefore, based on the results of previous studies and practical production benefits, this study recommends increasing the dietary proline supplementation starting from the early gestation period.

While this study provides a comprehensive exploratory analysis, several limitations must be acknowledged. First, the absence of direct measurements of placental Pro transport efficiency leaves open the question of whether Pro crosses the placenta intact or as metabolites like glutamate—a critical gap that could be addressed using isotope-labeled Pro tracing. In addition, for example, the LT-Pro group may improve mitochondrial function in offspring skeletal muscle. This finding is based on mRNA data, and further studies at the protein level are needed to confirm these potential mechanisms. Secondly, although the STAT3-CCND1 central signaling axis may be a key target for promoting muscle development in offspring, the potential epigenetic modifications (e.g., DNA methylation at *MYOD1* or *CCND1* loci) mediating Pro’s lasting effects remain unexplored. Single-cell epigenomic profiling of satellite cells could clarify these mechanisms. Finally, the lack of long-term follow-up on meat quality parameters (e.g., tenderness, water-holding capacity) limits our ability to fully assess the translational value of oxidative fiber programming. Future studies should prioritize longitudinal assessments of carcass traits at market weights and investigate cross-species conservation of Pro’s effects in other livestock models.

## Conclusion

This study redefines maternal Pro supplementation as a chrono-specific nutritional intervention that reshapes skeletal muscle development, likely through STAT3-CCND1-mediated proliferation, mitochondrial bioenergetic adaptation, and oxidative fiber specification. By identifying the G20–114 window as a period of heightened developmental sensitivity, we provide a roadmap for precision nutrition strategies in swine production. Our findings underscore the importance of developmental timing in nutritional interventions and provide a mechanistic foundation for precision feeding systems in livestock agriculture.

## Supplementary Information

Additional file 1: Table S1. The composition and nutrient levels of the diets (as-fed basis, %). Table S2. Primer sequences used for RT-PCR amplification.

## Data Availability

The datasets used during the current study are available from the corresponding author on reasonable request.

## Abbreviations

ATP: Adenosine triphosphate
BCAA: Branched-chain amino acid
CSA: Cross-sectional area
HE: Hematoxylin-eosin
LD: Longissimus dorsi
mTOR: Mechanistic target of rapamycin
MyHC: Myosin heavy chain
PM: Psoas major
Pro: Proline
SDH: Succinate dehydrogenase
TCA cycle: Tricarboxylic acid cycle
