# Supplementary material for: Proline-driven metabolic reprogramming promotes skeletal muscle hypertrophy and oxidative myofiber specification in porcine offspring: a stage-optimized maternal nutritional intervention
Source: J Anim Sci Biotechnol. 2025 Jul 15;16:99. doi: 10.1186/s40104-025-01232-7 (PMC12261827; doi:10.1186/s40104-025-01232-7)
Supplement: Supplementary file 1 — Additional file 1: Table S1. The composition and nutrient levels of the diets (as-fed basis, %). Table S2. Primer sequences used for RT-PCR amplification. [file 40104_2025_1232_MOESM1_ESM.docx]

**Table S1** The composition and nutrient levels of the diets (as-fed basis, %)^1^

| **Item** | **Diets**^2^ | |
| --- | --- | --- |
|  | **CON** | **Pro** |
| **Ingredients** |  |  |
| Corn | 29.08 | 29.04 |
| Soybean meal (43%) | 14.39 | 14.37 |
| Barley | 24.81 | 24.78 |
| Sorghum | 9.92 | 9.91 |
| Wheat bran | 9.92 | 9.91 |
| Soybean husk | 5.95 | 5.95 |
| Soybean oil | 0.99 | 0.99 |
| Mildewcide (50%) | 0.20 | 0.20 |
| Premix^3^ | 3.96 | 3.96 |
| Additional alanine | 0.78 | 0.39 |
| Additional proline |  | 0.50 |
| Total | 100.00 | 100.00 |
| **Calculated nutrient levels**^4^ | | |
| Dry matter | 88.72 | 88.62 |
| Crude protein | 14.55 | 14.53 |
| Crude fiber | 5.40 | 5.40 |
| Crude fat | 3.55 | 3.55 |
| Neutral detergent fiber | 16.77 | 16.75 |
| Net energy, kcal/kg | 2259.54 | 2256.87 |
| Starch | 40.17 | 40.12 |
| Lysine | 0.71 | 0.71 |
| Methionine | 0.24 | 0.24 |
| Threonine | 0.55 | 0.54 |
| Tryptophan | 0.16 | 0.16 |
| Total proline | 0.89 | 1.39 |
| **Analyzed nutrient levels**^5^ |  |  |
| Crude protein | 14.44 | 14.41 |
| Crude fiber | 5.90 | 6.00 |
| Neutral detergent fiber | 16.50 | 15.90 |

^1^Cited from https://doi.org/10.1016/j.aninu.2024.10.007

^2^CON = low proline level (0.89%) diet; Pro = proline level (1.39%) diet. The Pro levels in diets are expressed as totals

^3^Provided the following per kilogram of diets: VA 8000 IU, VD_3_ 1200 IU, VE 60 IU, VK_3_ 2 mg, VB_1_ 1 mg, VB_2_ 4 mg, VB_6_ 1 mg, VB_12_ 17 μg, folic acid 1.3 mg, biotin 0.2 mg, pantothenic acid 12 mg, choline 1 g, Cu 12 mg, Mn 25 mg, Zn 100 mg, Se 0.3 mg, I 0.4 mg

^4^Chemical concentrations were calculated using the values for feed ingredients from the National Research Council (2012) [15]

^5^Analyzed composition are shown as the mean values of two replicates

**Table S2** Primer sequences used for RT-PCR amplification

| Gene Name | Primer sequence (5′–3′) |
| --- | --- |
| β-actin | F: CACCTTCTACAACGAGCTGC  R: TCATCTTCTCACGGTTGGCT |
| *MSTN* | F: CAGTGATGGCTCCTTGGAAG  R: GAGTCTTGACGGGTCTCAGA |
| *CAT1* | F: TGCCCATACTTCCCGTCC  R: GGTCCAGGTTACCGTCAG |
| *CAT2* | F: GGGTCCGCGTATTTGTACAC  R: CTGCGAGGCCAGTGTAATTC |
| *EAAT3* | F: GACGTCACCCTGATCATTGC  R: GCAAAGGGGTTCACGATGTT |
| *B^O^AT1* | F: GCTCTGGTCGGTGCTCTTCT  R: CGTTTTGGGGATGACCTTG |
| *EAAT1* | F: GATGGGACCGCCCTCTAT  R: CGTGGCTGTGATGCTGATG |
| *LAT4* | F: CAGATCCAGAAGATCACCAAC |
|  | R: TGAAGGAGAGAATCTGTAGGG |
| *ASCT2* | F: CAAGATTGTGGAGATGGAGGAT |
|  | R: TTGCGAGTGAAGAGGAAGTAGAT |
| *GLAST* | F: CATGCCCATTCTTTCCTACCC |
|  | R: TTTCTTTCTTCGGAAAATGTTG |
| *LAT1* | F: GCCCATTGTCACCATCATC |
|  | R: GCCCATTGTCACCATCATC |
| *LAT2* | F: ACTACCTCTTCTATGGCATCAC |
|  | R: GCAAGTAGATGATGGGGAACAG |
| *MyHCI* | F: AGCCTCTTTCTTCTCCCAGGGACATTC |
|  | R: ATCCAGGCTGCGTAACGCTCTTTGAGGTTGTA |
| *MyHCIIx* | F: CTTTCCTCATAAAGCTTCAAGTTCTGCC |
|  | R: ATCCAGGCTGCGTAACGCTCTTTGAGGTTGTA |
| *MyHCIIb* | F: CATCTGGTAACATAAGAGGTACATCTAG |
|  | R: ATCCAGGCTGCGTAACGCTCTTTGAGGTTGTA |
| *MyHCIIa* | F: CATTGAGGCCCAGAATAGGC |
|  | R: TGCTTCCGTCTTCACTGTCAC |
| *FGF2* | F: GCAGAAGAGAGAGGGGTTGTGT |
|  | R: AAGCCAGTAATCTTCCATCTTCCTT |
| *TCF4* | F: ATCAGCAAGCACTGCCGACT |
|  | R: CCAACATTCCTCCGTAGCCA |
| *SIX1* | F: CAGCAGCCAGCACTACAGAG |
|  | R: GGCAGGGATGACCTTGTTTA |
| *MEF2D* | F: TCCGCTATATCGAAGGCCTG |
|  | R: GTCTGAGTCACCGCTGTAGT |
| *CCND1* | F: TGTTTGCAAGCAGGACTTTG |
|  | R: ACGTCAGCCTCCACACTCTT |
| *CCND2* | F: TGGGCTTCAGCAGGATGATG |
|  | R: ACGGAACTGCTGCAGGCTGT |
| *CCNB1* | F: CGGGATCCATGGCGCTCCGAGTCACCAG |
|  | R: CCGCTCGAGTTACACCTTTGCCACAGCCTTG |
| *MYF6* | F: TGCACCGGCTGGATCAG |
|  | R: CCGCACCCTCAAGATTCTCTT |
| *MYOG* | F: AACTACCTGCCCGTCCACCT |
|  | R: GTCCCCAGCCCCTTATCTTC |
| *MYOD1* | F: AGGGACAGGATAGAGCAGGG |
|  | R: TCAAATCTACGTCGCGGAGC |
| *IGF1* | F: GCCCAAGGCTCAGAAGG |
|  | R: TTTAACAGGTAACTCGTGC |
| *PAX7* | F: GTGCCCTCAGTGAGTTCGAT |
|  | R: TCCAGACGGTTCCCTTTGTC |
| *MYF5* | F: CCACGACTAACCCCAACCA |
|  | R: TTTTCCACCTGCTCCCTCA |
| *MEF2C* | F: ACCCCAAGACTGCTCACAA |
|  | R: GTTAGCTCAAACTCCACAT |
| *PPARGC1A* | F: GTGTCGCCTTCTTGTTCTTCTTTT |
|  | R: CGCATCCTTTGGGGTCTTT |
| *PPARGC1B* | F: AGAGGCACCCAGAGCGAAG |
|  | R: TTGTGGCATGCTGCAAATG |
| *MFN2* | F: GCCACACCACCAACTGCTTCC |
|  | R: TCTTGACGCTCCTCTTCTCCTCTG |
| *MFN1* | F: AGAAAGCACAAAGCACAGGGGATG |
|  | R: CACTGCTGACTGCGAGATACACTC |
| *MFF* | F: GACCTCCTCCAACCCCTCAAAATG |
|  | R: GACTAGCTGTCCATTCTGGCGAAC |
| *DNM1L* | F: TCTGAATCTGGTGGGCATGATTGC |
|  | R: CTCCGCAGTAAAGGACTCGAAGTG |
| *OPA1* | F: AGGCTCTTCAGGCTCGTCTCAAG |
|  | R: CGATTCTTCCAGTACAGCCACCTC |
| *SQSTM1* | F: AGCTGCCCTCAGCCCTCTCTA |
|  | R: GGCTTCTCTTCCCTCCATG |

*MSTN* = myostatin; *CAT1* = cationic amino acid transporter 1; *CAT2* = cationic amino acid transporter 2; *EAAT3* = excitatory amino acid transporter 3; *B^O^AT1* = broad neutral amino acid transporter 1; *EAAT1* = excitatory amino acid transporter 1; *LAT4* = L-type amino acid transporter 4; *ASCT2* = Alanine-Serine-Cysteine Transporter 2; *GLAST* = Glutamate Aspartate Transporter; LAT1 = L-type Amino Acid Transporter 1; *LAT2* = L-type Amino Acid Transporter 2; *MyHCI* = Myosin Heavy Chain I; *MyHCIIx* = Myosin Heavy Chain IIx; *MyHCIIb* = myosin heavy chain Ⅱb; *MyHCIIa* = myosin heavy chain Ⅱa; *FGF2* = fibroblast growth factor 2; *TCF4* = transcription factor 4; *SIX1* = sine oculis homeobox 1; *MEF2D* = myocyte enhancer factor 2d; *CCND1* = cyclin D1; *CCND2* = cyclin D2; *CCNB1* = cyclin B1; *MYF6* = myogenic factor 6; *MYOG* = Myogenin; *MYOD1* = Myogenic Differentiation 1; *IGF1* = Insulin-like Growth Factor 1; *PAX7* = Paired Box 7; *MYF5* = Myogenic Factor 5; *MEF2C* = Myocyte Enhancer Factor 2C; *PPARGC1A* = peroxisome proliferator-activated receptor gamma coactivator 1-alpha; *PPARGC1B* = peroxisome proliferator-activated receptor gamma coactivator 1-beta; *MFN2* = mitofusin 2; *MFN1* = mitofusin 1; *MFF*= mitochondrial fission factor; *DNM1L*  = dynamin 1 Like; *OPA1* = optic atrophy 1; *SQSTM1* = sequestosome 1
